# The Effects of Electrode Placement on Analgesia Using Transcutaneous Electrical Nerve Stimulation for Primary Dysmenorrhea: A Single-Blind Randomized Controlled Clinical Trial

**DOI:** 10.7759/cureus.39326

**Published:** 2023-05-22

**Authors:** Fabio Mendes Camilo, Paulo Sérgio Bossini, Patricia Driusso, Mariana Arias Ávila, Nivaldo Antônio Parizotto, Ueverton Rodrigues de Sousa, Rogério Rodrigo Ramos

**Affiliations:** 1 Department of Health Sciences, University Center of Santa Fé do Sul, Santa Fé do Sul, BRA; 2 Department of Biophotonics and Biomaterials, Institute of Research and Education in the Health Area (NUPEN), São Carlos, BRA; 3 Physical Therapy Post-Graduate Program, Federal University of São Carlos, São Carlos, BRA; 4 Physical Therapy Post-Graduate Program and Research Nucleus on Electrophysical Agents, Federal University of São Carlos, São Carlos, BRA; 5 Post-Graduate Program of Biotechnology on Regenerative Medicine and Medical Chemistry, University of Araraquara, Araraquara, BRA; 6 Department of Research, Faculty of Roseira (FARO), Roseira, BRA; 7 Department of Health Sciences, Brazil University, Fernandópolis, BRA

**Keywords:** physical therapy, analgesia, electrical stimulation, intermittent pain, menstrual cycle

## Abstract

Background

Primary dysmenorrhea (PD) refers to the occurrence of painful menstrual cramps without pathological involvement of the pelvic organs, with considerable morbidity and high prevalence among females of reproductive age.

Objective

The objective of this study is to present and test the efficacy of an innovative method of interactive transcutaneous electrical nerve stimulation (iTENS) for PD.

Methods and materials

This study is a single-blind controlled clinical trial. This was conducted at the outpatient clinic of the faculty of physical therapy. Females with PD (n=124) were divided into the treated group (transcutaneous electrical nerve stimulation {TENS} group {TG}, n=62) and the placebo group (PG, n=62). A single session of either iTENS or placebo intervention was used for 35 minutes. Pain, the duration of analgesia, and the use of pain medication were assessed before and after the intervention. Data from before and after the treatment were compared between groups (Student’s t-test). The level of significance was set at 5%.

Results

A significant decrease in pain (p<0.001) was observed after the intervention for the TG, with a more long-lasting analgesia (p<0.001) and decreased need for pain medication (p<0.001).

Conclusions

The proposed method of transcutaneous electrical nerve stimulation (TENS) application showed positive results for pain management on females with PD, with no reported adverse effects. The new proposed TENS application takes into account the preferences of the patient regarding positioning and the number of channels needed to cause analgesia. This application was able to promote almost complete analgesia in females with primary dysmenorrhea, and the analgesia persisted for more than one menstrual cycle.

## Introduction

Dysmenorrhea is one of the most common complaints of females of reproductive age [1] and refers to the occurrence of painful menstrual flow that, along with other symptoms, leads to considerable morbidity [2]. The prevalence of dysmenorrhea varies between 16% and 91% in adolescents and females of reproductive age, with severe pain in 2%-29% of cases [3,4], even causing absenteeism from school and work [5,6].

It is usually divided into primary dysmenorrhea (PD), characterized as pain during the menstrual period with no known pelvic pathology, and secondary dysmenorrhea, which is associated with some pathological conditions, such as endometriosis and adenomyoma [7,8]. Pain during PD usually lasts 8-72 hours [9] and is managed with analgesic or nonsteroidal anti-inflammatory drugs (NSAIDs), which are effective but can cause side effects [10,11].

The treatment for PD can be either pharmacological, nonpharmacological, and surgical [12], with the advantage of neuromuscular therapy not leading to adverse effects [13]. Another nonpharmacological approach used in PD is the use of electrical stimulation, such as transcutaneous electrical nerve stimulation (TENS), a noninvasive technique widely used to promote analgesia in acute and chronic health conditions [14,15]. It is easy to use, with no reported side effects [16,17], and its mechanism of action relies on the gate control theory and the activation of opioid receptors located in the central nervous system [18]. Studies [19,20] have shown that TENS can lead to a decrease in pain in those females suffering from PD. However, TENS, in a general manner, has been poorly used as the parameters used to improve pain symptoms in several kinds of pain [16,17] are not adequate, and data on optimal dosing, which include electrode positioning, are still not available [17]. For PD treatment, other studies have shown that TENS is effective in reducing pain [21-25], and a systematic review has shown evidence for that effect [11]. The parameters usually used include high frequencies (100-200 Hz) and should include adequate amplitudes [16] but do not take into account patients’ preferences and perceptions, which are key factors when considering the practical evidence-based physiotherapy [26,27].

Hence, the aim of the present study was to investigate a novel TENS protocol, namely interactive TENS (iTENS), for PD; this new protocol uses the participants’ preferences regarding electrode placement. The hypothesis was that iTENS would be effective in achieving complete analgesia, with promising results to PD treatment.

## Materials and methods

This is a single-blind randomized controlled clinical trial, with an allocation ratio of 1:1. This study was approved by the Ethics Committee of the Faculdades Integradas de Santa Fé do Sul (CEP-FISA/FUNEC, protocol number: 036/2010) and is registered at the ClinicalTrials.gov under the number NCT02205970. The participants gave their written and informed consent to take part in this study, which was conducted according to the 2008 Declaration of Helsinki. The study was conducted in the physical therapy section of the rehabilitation center.

To be included in the study, females should 1) be 18 years of age or older and be in the reproductive phase, 2) be nulliparous, 3) present PD symptoms in every menstrual cycle at least six months prior to the participation in the study, 4) experience pain considered moderate to severe (>40 mm in the visual analog scale {VAS}) during PD episodes, 5) be able to read and understand questionnaires in Portuguese, and 6) have undergone a general gynecological assessment within the past 18 months. The exclusion criteria were as follows: 1) the medical diagnosis of secondary dysmenorrhea, 2) not having regular menstrual cycles (typically 21-35 days [28]) that could be related to gynecological disorders [29], 3) low-intensity (VAS<40 mm) pain during PD episodes or episodes in nonsequential menstrual cycles, 4) the use of intrauterine devices or pacemakers, 5) the use of oral contraceptives, 6) the diagnosis of a serious condition (such as a malignant tumor, cardiovascular disease, and metastasis) or cognitive deficits that prevented the participants to understand the evaluation procedures, 7) drugs or alcohol addiction, and 8) the use of analgesics 48 hours prior to the evaluation session.

The sample size was based on Dworkin et al. [30], considering a clinically significant difference in the visual analog scale (VAS) intensity of 20%. The sample size was determined using the G*Power software (Heinrich Heine University Düsseldorf, Düsseldorf, Germany), considering a significance level of 0.05 and a power of 0.80 to obtain an effect size of 0.15. Based on these criteria, at least 120 participants (60 in each group) were necessary. The participants who filled the inclusion criteria and were willing to take part in the study were randomly assigned into two groups: interactive TENS (iTENS) or placebo. Randomization was performed through a website (www.randomization.com) and placed accordingly in sealed opaque envelopes that were picked up by each participant after initial evaluation. This sealed envelope was given to the physical therapist responsible for the treatment and blinded for the evaluation results, and the participant did not see to which group she was allocated.

Experimental design

All participants were invited for an initial evaluation that occurred in a day that was not part of the menstrual period. This initial evaluation, performed by a researcher blinded to the participants’ allocation, aimed to collect demographic data, as well as to check the inclusion and exclusion criteria. After that, they were instructed to contact the main researcher as soon as they started feeling pain due to the start of the menstrual period, and they received the intervention up to six hours after the pain onset. They were also instructed not to take any kind of pain medication before the intervention. Then, the participants received the intervention according to the opaque sealed envelope they received during the initial evaluation, either iTENS (TENS group, TG) or placebo treatment (placebo group, PG). The participants from both groups underwent one single treatment session on one single menstrual cycle, on the day they started experiencing PD pain. All of them answered the VAS for pain immediately before and after the intervention and reported the time it took for the painful sensation to start again after the intervention; also, they reported if they needed any kind of painkillers or nonsteroidal anti-inflammatory drugs (NSAIDs) after pain onset.

Outcomes

Prior to any procedure, the participants underwent an initial evaluation, on a single day different from the treatment day, in which clinical, demographic, and PD data were collected. Afterward, to assess pain intensity, a 100 mm VAS was used. A change of 20 mm was considered clinically relevant [30,31]. The duration (in hours) of the analgesia, the use of analgesics after the intervention, and the medication effectiveness were also registered.

Intervention

Each treatment session lasted for 35 minutes in which the participants were grouped in either the TG or PG. The description of the electrotherapy parameters follows Barbosa et al. [32]. A NeuroDyn portable (IBRAMED, São Paulo, Brazil) with two-channel device was used. Its current has square biphasic asymmetric waveform. Electrodes (50×50 mm) were of carbon-impregnated silicone rubber and were placed with hypoallergenic gel and strapped (after finding the correct position) with micropore adhesive tape. The participants were instructed to lie down in ventral decubitus in the most comfortable way they could and to keep the thoracic and lumbar regions exposed (for the best electrode placement). Afterward, the intervention (either iTENS or placebo) was applied. Current parameters for the TG were 400 µs of pulse duration, 120 Hz of frequency, and amplitude enough to cause a strong paresthesia (sensory level, with no motor response). The participants from the TG received a 35-minute application during a single menstrual cycle.

To find the best electrode placement, once the participants were lying in ventral decubitus, the electrodes were placed on the paravertebral lumbar column at the level of L3/L4, which is the spinal level that receives nociceptive information from the uterus [33]. The current was applied, and the participant was questioned on her pain. If she reported any, the mapping of the area was performed, and the electrodes were moved, one at a time, to try to find one positioning in which the participant reported a strong paresthesia rather than pain. The researcher randomly chose one of the electrodes and started moving it without any specific standard direction, in low speed. At each move, the participant was questioned about her pain, and when she reported a decrease in pain, the researcher kept that electrode in that site and started moving the other electrode, corresponding to the same channel. The sweeping of the area continued until the participant described full analgesia. After locating the best spots for positioning the electrodes, if full analgesia for the dysmenorrhea was not obtained, the researcher changed the initial current parameters, according to the following sequence: increasing amplitude (bearable limit), adjusting frequency, readjusting electrode positioning, or including another channel. Those data were registered.

Once the desired analgesia was obtained, the electrodes were fixed, and the application time was 35 minutes. Every five minutes during the application time, the participant was questioned about current amplitude, and whenever needed, it was increased up to the moment the participant reported, again, strong paresthesia and not pain. A third researcher (who did not take part in the research) was responsible for recording the final VAS.

The participants from the PG underwent a similar application protocol, with electrodes fixed over the L3/L4 region with micropore adhesive tape and active electrical current application (in which the participants referred strong but comfortable paresthesia) for only one minute, after which the amplitude was quickly and gradually reduced to zero. The simulated application lasted for 35 minutes. Before and after the application, the participants answered the VAS for pain. The participants from this group were asked every five minutes about comfort with no mention of TENS. If a participant asked about the lack of sensation from the TENS application, the responsible researcher answered the question in a general manner, without giving clues about placebo.

Data analysis

Statistical analysis was performed with Statistical Package for Social Sciences (SPSS) version 20 (IBM SPSS Statistics, Armonk, NY). Data are presented as mean±standard deviation for data with normal distribution according to Shapiro-Wilk test. Qualitative data are presented as number of females (percentage). To compare the effects before and after the treatment in both groups, paired t-test was used. Independent t-test was applied to compare the effects of interactive TENS to placebo. Chi-square test was used to determine the difference of the groups on qualitative variables. Effect sizes for all quantitative variables were measured with the Cohen’s d coefficient after the intervention. An effect size greater than 0.8 was considered large, around 0.5 moderate, and less than 0.2 small [34]. The significance level was set at 5%.

## Results

**Figure 1:**
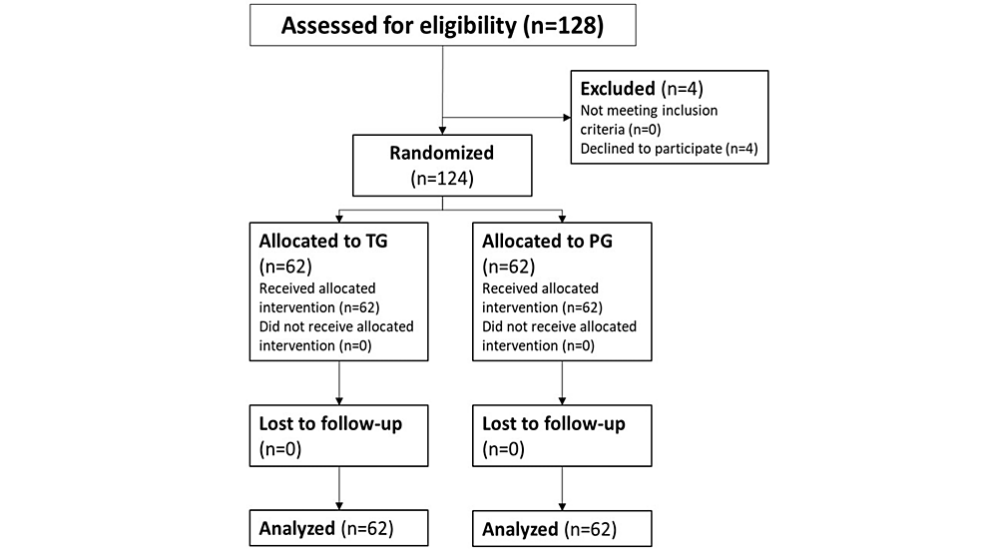
Flowchart TG, TENS group; PG, placebo group; TENS, transcutaneous electrical nerve stimulation

This study was conducted from January 2012 to December 2013. For the first stage, 128 females were eligible, and 124 took part in the study. Four participants declined to participate. Figure 1 shows the study’s flowchart.

The participants (n=124) were homogeneous in terms of age, pain intensity before the treatment, and the use and efficacy of pain medication (Table 1). The participants from PG used only one channel and reported mild analgesia (from 7.65±1.28 to 7.00±1.36; p=0.007) after the intervention (Table 2). The participants from the TG used either one (n=59, 95.2%) or two (n=3, 4.8%) channels and reported significant analgesia (from 7.58±1.36 to 0.01±0.05; p<0.001) that lasted more than eight hours (Table 2). Of those females on the TG, only 4.8% needed adjustments of amplitude and frequency (that were increased by 20%), and at last, the researcher included another channel so that the analgesia would be obtained. When the second channel was included, amplitude and frequency were adjusted again to the initial levels and accordingly.

**Table 1:** Volunteers’ characteristics before the intervention (n=124) Data are presented as average±standard deviation or the number of cases TG, TENS group; PG, placebo group; TENS, transcutaneous electrical nerve stimulation

Characteristics of the volunteers	TG (n=62)	PG (n=62)	P value
Age (years)	22.5±4.3	22.9±4.5	0.50
Pain intensity before treatment (cm)	7.6±1.4	7.6±1.3	0.20
Use of medication			
Yes	60	59	0.85
No	2	3	
Medication effectiveness			
Effective	3	2	0.50
Relative	48	50	
Ineffective	9	7	

**Table 2:** Results after the intervention (n=124) Data presented as mean±standard deviation TG, TENS group; PG, placebo group; CI, confidence interval; NA, not applicable

Variables	TG (n=62)	PG (n=62)	Difference (PG-TG) (95% CI)	P value	Cohen’s d (95% CI)
Pain intensity after intervention (cm)	0.01±0.05	7.00±1.36	6.99 (6.65, 7.33)	<0.001	-7.26 (-8.18, -6.25)
Analgesia duration (hour)	8.44±0.07	0.37±0.07	-8.07 (-8.09, -8.05)	<0.001	16.94 (14.71, 18.96)
Pain medication use after intervention (n)	17	59	NA	<0.001	NA

## Discussion

The results of the present study show important improvement in pain experienced by females with PD by using the iTENS method. TENS already presents evidence of analgesic results for dysmenorrhea [8,19,35,36], especially when adequate parameters are used [16,17]. The main purpose of the present study, however, was to test if positioning electrodes according to the participant report of complete analgesia was possible and feasible as a way to optimize TENS analgesia for those females.

The results obtained showed a significant pain intensity reduction after the use of iTENS, with no adverse effects. The improvement achieved was greater than the minimal important clinical difference (of 2 cm in the VAS, according to Dworkin et al. [30]). In the present study, the difference observed in VAS (before and after the application) was 7.57 (±1.36) points in the active and 0.65 (±0.83) points in the control group, with 93% of the TG reporting complete analgesia after one single session. Those results account positively for the iTENS method to treat PD symptoms. In the studies by Proctor et al. [15] and da Silva Paulino et al. [35] using TENS for PD symptom control, the results showed the benefit of TENS over placebo, with pain relief. The results from the present study show a complete remission of pain after treatment, with results lasting for more than eight hours after the application, and a decrease in the use of pain medication to control PD symptoms.

The difference of the proposed method relies on the adequateness of the electrical current parameters, with strong yet tolerable current amplitude and adequate pulse duration (400 µs), as well as considering the electrode positioning according to the participant’s report. The so-called conventional or high-frequency stimulation was used in all revised studies with short-pulse duration and low amplitude [35]. However, some authors state that it is important to adjust the amplitude of the use during TENS application in order to obtain maximum analgesic effect [14,16,17,37-39]. Based on this context, during the application of iTENS, the intensity remained always in maximum tolerable levels, causing strong paresthesia. Increasing the amplitude along the application of TENS may have contributed to achieving the level of analgesia observed in the results of the present study, which corroborates previous studies that showed that when TENS is applied in adequate intensities, it produces greater hypoalgesia [37,38].

The placebo group presented a mild reduction in painful symptoms after the placebo TENS application. Even though this was expected [40-42], the magnitude of pain reduction of PG (0.65) was lower than the minimal clinically important difference considered [30,31] and did not last long (22 minutes). Also, the need for pain medication was significantly reduced for the TG, as 95% of participants allocated to the PG needed medication while 27% of participants of the TG needed analgesic medication after intervention. Given that the healthcare costs of females with PD may be 2-3 times higher than females without PD [43], reducing the need for medication may be one goal of PD treatment, which was successfully achieved by the TG in the present study.

The present study has some limitations. No follow-up was performed, which may weaken more incisive conclusions on the efficacy of the method. Other measures could have been taken, such as pain sensitivity (with pressure pain threshold measurement), quality of life, and sleep quality. Given that the method was firstly proposed in the present study, it was not compared to active TENS or other known analgesic nonpharmacological method, which should be the object of further studies.

## Conclusions

The proposed method of TENS application showed positive results for pain management in females with PD, with no reported adverse effects. The new proposed TENS application takes into account the preferences of the patient regarding positioning and the number of channels needed to cause analgesia. This application was able to promote almost complete analgesia in females with primary dysmenorrhea.
